# Therapeutic Potential of Big-Belly Seahorse Derived Peptide in Blood Pressure Regulation and Protection Against Aortic, Renal, and Cardiac Injuries on Spontaneously Hypertensive Rats

**DOI:** 10.3390/pharmaceutics17111449

**Published:** 2025-11-10

**Authors:** Hyo-Geun Lee, Habaragoda Dewage Tharushi Udayangani Wijerathne, Taeho Kim, Si-Hyeong Park, Won-Kyo Jung, Jae-Young Oh, Mi-Jin Yim, Jeong Min Lee, Seok-Chun Ko, Dae-Sung Lee, Hyun-Soo Kim

**Affiliations:** 1 National Marine Biodiversity Institute of Korea, 75, Jangsan-ro 101-gil, Janghang-eup, Seocheon 33362, Republic of Korea; hyogeunlee92@mabik.re.kr (H.-G.L.); mjyim@mabik.re.kr (M.-J.Y.); lshjm@mabik.re.kr (J.M.L.); seokchunk@mabik.re.kr (S.-C.K.); 2 Department of Seafood Science and Technology, Institute of Marine Industry, Gyeongsang National University, 2-9, Tongyeonghaean-ro, Tongyeong-si 53064, Republic of Korea; tudayangani14@gmail.com (H.D.T.U.W.); qkrtlgud87@naver.com (S.-H.P.); 3 Jeju Bio Research Center, Korea Institute of Ocean Science and Technology, Jeju 63349, Republic of Korea; kt1024@kiost.ac.kr; 4 Major of Biomedical Engineering, Pukyong National University, 45, Yongsoro, Namgu, Busan 48513, Republic of Korea; wkjung@pknu.ac.kr; 5 Food Safety and Processing Research Division, National Institute of Fisheries Science, 216, Gijanghaeanro, Gijangeup, Busan 46083, Republic of Korea; ojy0724@korea.kr

**Keywords:** *Hippocampus abdominalis*, bioactive peptide, angiotensin Ⅱ, angiotensin-converting enzyme 2

## Abstract

**Background/Objectives:** Marine-derived bioactive peptides have been reported to possess blood pressure-regulatory effects. However, most studies have focused on the antihypertensive effects after single-dose administration, and research on long-term administration and its protective effects against hypertension-induced tissue damage remains limited. Therefore, this study aimed to investigate the long-term antihypertensive efficacy of IGTGIPGIW, a bioactive peptide derived from *Hippocampus abdominalis* (*H. abdominalis*), and its protective effects on hypertension-related tissue damage. **Methods:** To evaluate the blood pressure-regulatory effects, spontaneously hypertensive rats (SHRs) were orally administered a high-dose (50 mg/kg) IGTGIPGIW peptide group (H-IGTGIPGIW) for 8 weeks. Systolic blood pressure (SBP), diastolic blood pressure (DBP), and mean arterial pressure (MAP) were monitored weekly. Serum levels of angiotensin II (Ang II), angiotensin-converting enzyme (ACE), and angiotensin-converting enzyme 2 (ACE2) were measured to assess the peptide’s regulatory effects on the renin–angiotensin system. Histological analyses of the aorta and heart tissues were performed to evaluate the protective effects against hypertension-induced tissue damage. **Results:** After 8 weeks of treatment, H-IGTGIPGIW significantly reduced SBP, DBP, and MAP compared with SHRs. Serum Ang II and ACE levels were significantly decreased, while ACE2 levels were significantly increased. Histological analyses demonstrated that IGTGIPGIW alleviated aortic wall thickening and reduced renal and cardiac tissue damage in SHR. **Conclusions:** IGTGIPGIW, a bioactive peptide derived from *H. abdominalis*, effectively regulated blood pressure by modulating serum Ang II, ACE, and ACE2 levels. Moreover, it protected against hypertension-induced aortic, renal and cardiac tissue damage, suggesting its potential as a functional ingredient for managing hypertension.

## 1. Introduction

Hypertension is defined as a chronic condition in which systolic blood pressure (SBP) and/or diastolic blood pressure (DBP) remain persistently above 130/80 mmHg [1]. Hypertension is a globally prevalent public health problem. An estimated 1.28 billion adults aged 30–79 years old have hypertension worldwide, most two/thirds hypertension patients living in low and middle-income countries [2]. According to the WHO, hypertension ranks fifth among the leading causes of death worldwide. Furthermore, 46% of adults didn’t recognize hypertension, and less than half of adults recognized hypertension; 42% of hypertension patients were diagnosed and treated, and 21% of the population have it under control. Hypertension is recognized as a major risk of cardiovascular disease, stroke [3], myocardial infarction, coronary heart disease, stroke, kidney failure, heart failure, and vascular dementia [4]. Therefore, careful monitoring of symptoms such as headache, neck heaviness, and nosebleeds is urged to avoid hypertension-associated complications [5].

To control elevated blood pressure and prevent hypertension-related complications, regulation of the renin–angiotensin system (RAS) pathway is necessary, and ACE inhibitors offer a potentially effective approach for managing blood pressure in HTN. Therefore, some previous research has put forth new antihypertensive therapies. Recently, Chun et al. (2020) [6] reviewed a new strategy for vasorelaxation, and Kuang et al. (2020) [7] suggested a combination of antihypertensive and lipid-lowering therapies. Several types of antihypertensive agents have been developed to prevent hypertension-related complications. Among the synthetic antihypertensive agents, thiazide diuretics are the most commonly used monotherapies. Synthetic antihypertensive agents have known side effects, including hypokalemia, hyponatremia, hypercalcemia, hyperglycemia, hyperuricemia, hyperlipidemia, metabolic alkalosis, and sulfonamide allergy [8]. Due to the negative side effects associated with synthetic agents, numerous researchers are focusing on creating natural antihypertensive agents that are safe for the human body.

Marine bioresources have been reported to possess valuable biological potential, including antioxidant, anti-inflammatory, anticancer, anti-obesity, antidiabetic, and antihypertensive activities. Recent trends have focused on investigating marine-derived natural products [9]. Following previous publications [10] reported that if marine bioresources are sustainably exploited, they could be applied in the production of various bulk commodities such as marine-derived biomaterials, biofuels, and bioplastics. In addition, the overconsumption and depletion of bioresources have led to the utilization of byproducts from food and bioprocessing industries [11,12,13,14,15]. Kim et al. reviewed the importance of by-products from fish processing, including collagen and gelatin from fish skin, fish oil, fish bones, and fish organs [16]. They also emphasized in their study the utilization of byproducts from fish processing industries. Marine fish are known sources of valuable bioactive peptides, and numerous studies have explored enzymatic hydrolysates and their bioactive peptides [17]. In particular, large-scale antihypertensive activities of fish hydrolysates and their peptide have been reported [18,19,20,21], as have fish proteins [22]. In contrast, a considerable portion of antihypertensive studies have been dedicated to evaluating the short-term or single-dose effects of angiotensin converting enzyme (ACE) inhibition [23,24,25]. Therefore, a new strategy and therapy are needed to improve hypertension and its related comorbidities, including stroke, type 2 diabetes, obesity, hyperlipidemia, and metabolic syndrome [26,27,28]. Bioactive peptides have attracted considerable attention as potential natural agents for blood pressure regulation due to their strong blood pressure regulatory effects. In our previous study, we found that a peptide derived from *Hippocampus abdominalis* (*H. abdominalis*) inhibited angiotensin-converting enzyme (ACE) activity and exhibited a single-dose blood pressure-lowering effect in spontaneously hypertensive rats (SHR) [29]. However, further studies are needed to evaluate the long-term antihypertensive effects of this peptide and to investigate its potential protective roles against hypertension-induced organ damage.

Throughout this study, we have focused on assessing the blood pressure-regulatory effects and protective effects on hypertensive-induced tissue damage of the peptide IGTGIPGIW from *H. abdominalis* in vivo SHR. Our research highlights the potential of the big-belly seahorse-derived peptide IGTGIPGIW as an important preliminary study for the development of functional foods targeting blood pressure regulation and protection against hypertension, aortic, renal, and cardiac injuries.

## 2. Materials and Methods

### 2.1. Materials

Serum ACE, Ang II, and ACE 2 were analyzed using commercial analysis kits from LS Bio (Washington, DC, USA). DPX mounting solution (Sigma Aldrich, St. Louis, MO, USA) and images of the stained tissues were captured using an optical microscope fitted with a Cool SNAP-Pro color digital camera (Olympus, Tokyo, Japan).

### 2.2. Peptide Synthesis

The peptide IGTGIPGIW (Molecular weight 913 Da) was synthesized by Anygen (Anygen Inc., GwangJu, Korea) based on its mass and sequence, and used for the subsequent in vivo experiments. The peptide was purified by reverse-phase high-performance liquid chromatography using a HPLC system (Shimadzu HPLC Lab Solution, Tokyo, Japan) equipped with a SHIMADZU C18 analytical column. The elution was carried out using a linear gradient of 5–65% buffer B (0.05% trifluoroacetic acid in acetonitrile) over 30 min, with buffer A consisting of 0.05% trifluoroacetic acid in water. The flow rate was 1.0 mL/min, the detection wavelength was 230 nm, and the column temperature was maintained at 35 °C. The purity of the synthesized peptide was determined to be 86.3%.

### 2.3. Animal Care and Experimental Treatment

Twenty-five spontaneously hypertensive rats (SHR) were acquired from Jung Ang Lab Animal, Inc. (Seoul, South Korea). The Institutional Animal Care and Use Committee at Jeju National University authorized the use of 4-week-old female SHR for the experiments. The rats were kept in controlled environmental conditions with a temperature range of 20–22 °C, 55% humidity, and a 12-h light-dark photoperiod. Following a one-week acclimatization period, the animals were randomly assigned to five groups (*n* = 5 per group) and received different treatments. The experimental groups consisted of a WKY control group and an SHR control group, both administered drinking water; a low-dose (25 mg/kg) IGTGIPGIW peptide group (L-IGTGIPGIW); a high-dose (50 mg/kg) IGTGIPGIW peptide group (H-IGTGIPGIW); and a group treated with 100 mg/kg sardine-derived peptide (SP). All treatments were delivered orally at a volume of approximately 500 μL each morning. All experiments were conducted in accordance with the animal ethical guidelines established by the Animal Care Center at Jeju National University (2017-0017).

### 2.4. Measurement of SBP, DBP, MAP

To determine the antihypertensive effects of the peptide purified from *H. abdominalis*, blood pressure measurements were taken in SHR aged 7–8 weeks and weighing 250–300 g. The SBP and DBP were recorded using a tail-cuff volume pressure system (CODA, Kent Scientific, Torrington). Measurements were taken every 3 h between 15:00 and 03:00 over a 12 h period. For each time point, three independent measurements were averaged, and the results were displayed as graphs. Additionally, mean arterial pressure (MAP) was calculated using the following formula.

$$Map\left(mmHg\right)=DBP+\frac{SBP-DBP}{3}$$

### 2.5. Blood Biochemical Assay for Serum ACE, Ang Ⅱ, ACE2

Blood samples were collected from the inferior vena cava using ethylenediaminetetraacetic acid-rinsed syringes. The collected blood samples were transferred to heparin-coated tubes in a 4 °C maintained refrigerator. The tubes were then centrifuged at 4 °C and 1000 rpm for 10 min to clarify the serum for biochemical analysis. Following the centrifuge process, the supernatant was carefully separated and preserved in a freezer at −80 °C for future use. Colorimetric ELISA kits were applied to measure the serum levels of ACE, Ang II, and ACE2 (LS-bio, Washington, DC, USA).

### 2.6. Histology and H&E Staining

Histological analysis was performed on hypertension-induced aortic, renal, and myocardial tissue damage in the SHR. The target tissues were first isolated and then fixed in a 10% formalin solution. Following fixation, the tissues underwent dehydration through an automatic tissue processor and were subsequently embedded in paraffin wax. After dehydration, the tissues were embedded in paraffin blocks, which were then sectioned into thin slices of 5 µm. These slices were placed on slides and left to dry at 37 °C for 24 h. After 24 h, the slides were treated with 100% xylene for deparaffinization, stained with hematoxylin and eosin, and then washed three times with DW. Finally, DPX mounting solution (Sigma Aldrich, St. Louis, MO, USA) was utilized to mount the slides. Images of the stained tissues were captured using an optical microscope fitted with a Cool SNAP-Pro color digital camera (Olympus, Tokyo, Japan).

### 2.7. Statistical Analysis

All of the measurements were analyzed in independent triplicate and presented as the mean ± standard deviation (SD). Statistical analyses were performed using one-way ANOVA with Dunnett’s post-hoc test in GraphPad Prism (Version 9.4.1; GraphPad Software Inc., San Diego, CA, USA). P-values (* *p* < 0.05, ** *p* < 0.01, *** *p* < 0.001, and **** *p* < 0.0001 compared with the SHR control, # *p* < 0.05, ## *p* < 0.01, ### *p* < 0.001, and #### *p* < 0.0001 compared with the WKY control) of <0.05 were considered significant.

## 3. Results

### 3.1. Blood Pressure Regulatory Effect of Long-Term IGTGIPGIW Administration in SHR

**Figure 1:**
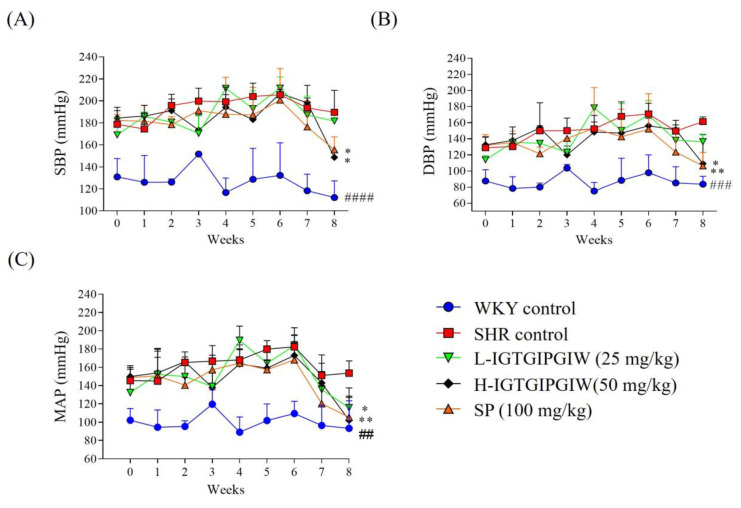
Blood pressure regulatory effects of IGTGIPGIW in spontaneously hypertensive rats (SHR). SBP (**A**), DBP (**B**) and MAP (**C**) in the SHR during 8 weeks of IGTGIPGIW administration. WKY control, Wistar Kyoto rats; SHR control, spontaneously hypertensive rats; SP, SHR treated with 100 mg/kg of sardine peptide; L-IGTGIPGIW, SHR treated with 50 mg/kg of IGTGIPGIW; H-IGTGIPGIW, SHR treated with 100 mg/kg of IGTGIPGIW. The thickness of the aorta was measured using ImageJ software (v.1.8.0). Data expressed as the mean ± SD. Significant differences were identified at * *p* < 0.05 and ** *p* < 0.01, as compared to the SHR control; ## *p* < 0.01, ### *p* < 0.001, and #### *p* < 0.0001 as compared to the WKY control.

Long-term regulatory effects of the peptide IGTGIPGIW on SBP, DBP, and MAP were observed over eight weeks in spontaneously hypertensive rats (SHR). Blood pressure measurements were taken using a noninvasive tail-cuff CODA system, and the SBP, DBP, and MAP results are presented in Figure 1. At the beginning of the experiment, the SBP, DBP, and MAP levels in SHR control were markedly elevated compared with those of WKY control. Specifically, baseline SBP, DBP, and MAP levels in SHR were approximately SBP (178.83 ± 15.38 mmHg), DBP (126.77 ± 12.75 mmHg), and MAP (145.61 ± 14.18 mmHg), indicating hypertension compared to WKY control which showed SBP (130.99 ± 16.71 mmHg), DBP (85.21 ± 13.71 mmHg), and MAP (102.03 ± 12.75 mmHg). During the 8-week experimental period, weekly blood pressure measurements revealed that the SHR control exhibited SBP levels ranging from 180 to 200 mmHg, DBP from 120 to 170 mmHg, and MAP from 140 to 180 mmHg. In contrast, the WKY control group showed SBP values ranging from 110 to 150 mmHg, DBP from 70 to 100 mmHg, and MAP from 80 to 120 mmHg. Notably, in the IGTGIPGIW-treated group, blood pressure did not show a significant decrease during the 6 weeks of treatment, but a gradual, though not statistically significant, reduction was observed from week 7. However, at week 8, SBP, DBP, and MAP levels in the IGTGIPGIW-treated group were significantly reduced compared to those in the SHR control group, indicating the antihypertensive effect of the peptide. Our research findings demonstrated that the final blood pressure levels in the L-IGTGIPGIW group were DBP (136.55 ± 9.26 mmHg) and MAP (115.80 ± 21.74 mmHg), indicating a significant reduction compared to the SHR control. In contrast, the H-IGTGIPGIW group exhibited a significant decrease in SBP (148.72 ± 5.26 mmHg), DBP (109.14 ± 35.87 mmHg), and MAP (102.01 ± 25.06 mmHg). Similarly, the SP-treated group showed markedly reduced blood pressure values, with SBP (155.63 ± 11.71 mmHg), DBP (106.25 ± 16.82 mmHg), and MAP (105.41 ± 23.08 mmHg). These findings suggest that long-term administration of IGTGIPGIW effectively lowers blood pressure in SHR, with the H-IGTGIPGIW showing the most pronounced antihypertensive effects.

### 3.2. Effect of IGTGIPGIW on Serum ANG and ACE Levels in SHR

The blood pressure regulatory effects of IGTGIPGIW were confirmed by measuring serum Ang-ACE, a major component of the renin-angiotensin system (RAS). Serum Ang-ACE profiling is presented in Table 1. Initial serum profiling demonstrated that the serum levels of Ang Ⅱ, ACE, and ACE2 in the SHR control were Ang Ⅱ (1323.20 ± 15.76 pg/mL), ACE (9.46 ± 0.12 ng/mL), and ACE2 (0.70 ± 0.03 ng/mL), respectively. In contrast, the WKY control group exhibited significantly lower levels of Ang II (1155.25 ± 48.04 pg/mL), ACE (8.62 ± 0.02 ng/mL), and ACE2 (0.45 ± 0.07 ng/mL) compared to the SHR group. However, after 8 weeks of IGTGIPGIW treatment, Ang II and ACE levels were significantly reduced, whereas ACE2 levels were significantly elevated compared to the SHR group. Our research findings demonstrated that the serum ACE level in the L-IGTGIPGIW-treated group was significantly elevated, reaching approximately ACE (8.88 ± 0.42 ng/mL) and the H-IGTGIPGIW-treated group exhibited decreased serum levels of Ang II (1209.75 ± 6.36 pg/mL), ACE (8.66 ± 0.32 ng/mL), and increased ACE2 (0.88 ± 0.02 ng/mL). Similarly, the SP-treated group also showed elevated levels of Ang II (1178.37 ± 9.93 pg/mL), ACE (8.63 ± 0.08 ng/mL), and ACE2 (0.96 ± 0.02 ng/mL) in the serum. These results suggest that IGTGIPGIW exerts its blood pressure-lowering effects by regulating serum Ang-ACE levels, thereby modulating the renin-angiotensin system (RAS) and ultimately influencing blood pressure in SHR.

### 3.3. Protective Effect of IGTGIPGIW on Hypertension-Induced Aortic Wall Damage in SHR

**Figure 2:**
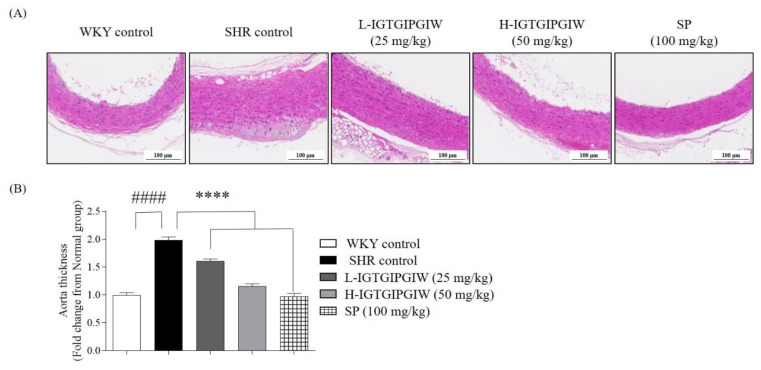
Effect of IGTGIPGIW on Patho morphology of the aorta in spontaneously hypertensive rats (SHR). SHR at 8 weeks showing abnormal changes such as medial and blood vessel hypertrophy in the aorta. Histologic H&E staining of the aorta in SHR (**A**) and aorta diameters (**B**). Data expressed as the mean ± SD. Significant differences were identified at **** *p* < 0.0001, as compared to the SHR control; #### *p* < 0.0001, as compared to the Wistar Kyoto rats (WKY) control.

To investigate the protective effect of IGTGIPGIW on aortic wall damage induced by hypertension in SHR, we conducted histological analysis of the aorta obtained from SHR. Persistent high blood pressure leads to thickening of the aortic wall, which in turn contributes to a vicious cycle of elevated blood pressure. Therefore, we captured the aorta tissue and measured aortic wall thickness to further assess the protective effect of IGTGIPGIW on structural damage induced by hypertension. Aortic wall thickness of each group is presented in Figure 2. Our research finding shows that the aortic wall thickness abnormally increased, and the irregular structure of aortic tissue was captured in SHR control. While the dense and normal aortic wall tissue is captured in the WKY control. In addition, the aorta tissues of IGTGIPGIW- and SP-treated group exhibited dense and structurally normal, similar to WKY control (Figure 2A). Figure 2B shows that the aortic wall thickness significantly increased by 1.98 ± 0.06-fold in the SHR control compared with the WKY control group, 0.91 ± 0.04-fold. However, aortic wall thickness was significantly reduced following IGTGIPGIW treatment. The L-IGTGIPGIW-treated group exhibited a 1.61 ± 0.03-fold thickness, and the H-IGTGIPGIW-treated group showed a 1.16 ± 0.04-fold thickness. Similarly, the SP-treated group showed a 0.98 ± 0.05-fold aortic wall thickness. These results indicate that IGTGIPGIW may protect against hypertension-induced aortic damage.

### 3.4. Protective Effect of IGTGIPGIW on Hypertension-Induced Kidney Damage in SHR

**Figure 3:**
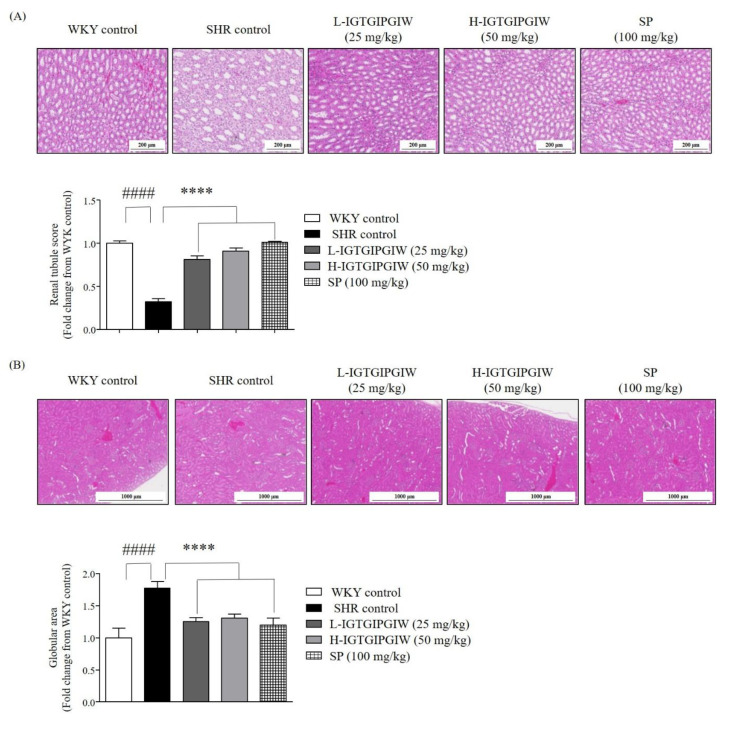
Effect of IGTGIPGIW on Patho morphology of the renal tubule and renal glomeruli in spontaneously hypertensive rats SHR. SHR at 8 weeks showing abnormalities in the renal tubule and glomeruli. Damaged renal tubules were integrated and collapsed, and hypertrophied glomeruli were observed in SHR. Histologic H&E staining of renal tubule (**A**) and renal glomeruli (**B**). The area of the renal tubule and glomeruli was measured using ImageJ software (v.1.8.0). Data expressed as the mean ± SD. Significant differences were identified at **** *p* < 0.0001, as compared to the SHR control; #### *p* < 0.0001, as compared to Wistar Kyoto rats (WKY) control.

To extend our investigation beyond vascular protection, we examined whether IGTGIPGIW also exerts protective effects against hypertension-induced kidney damage in SHR. The kidney is highly susceptible to sustained microvascular hypertension, which can result in glomerular and tubular structural damage. Histological examination was therefore performed on kidney tissues collected from SHR to evaluate potential morphological changes. Particular attention was given to the structural integrity of renal tubules and glomeruli as indicators of hypertensive damage. In Figure 3A, the renal tubule images reveal distinct structural differences between the SHR control and WKY control groups. In the SHR control group, the renal tubules appeared enlarged, sparsely distributed, and reduced in number. In contrast, the WKY control group exhibited densely arranged renal tubules with relatively higher numbers. Similarly, the IGTGIPGIW and SP-treated groups also displayed densely arranged renal tubules with a relatively higher number, resembling the morphology observed in the WKY control group. To evaluate renal tissue damage, we measured both the number of renal tubules. The renal tubule number significantly decreased by 0.32 ± 0.04-fold in the SHR control compared with the WKY control, 1.00 ± 0.02-fold. However, renal tubule number remarkably increased with IGTGIPGIW treatment. The L-IGTGIPGIW-treated group significantly increased renal tubule number by 0.81 ± 0.05-fold. Furthermore, H-IGTGIPGIW treatment significantly reduced the renal tubule number by 0.91 ± 0.03-fold. Similarly, SP treatment increased the renal tubule number significantly. Figure 3B shows the glomerulus in the kidney. The kidney glomerulus appears enlarged and more spread in the SHR control. The glomerulus in the WKY control was densely arranged and compact in structure. Similarly, the glomeruli in the IGTGIPGIW and SP-treated groups also exhibited a morphology similar to that seen in the WKY control group. To investigate kidney tissue damage, we measured the size of the glomeruli in the kidney. The glomerulus size significantly increased by 1.77 ± 0.10-fold in the SHR control compared with WKY control 1.00 ± 0.15-fold. The glomerulus size remarkably decreased with IGTGIPGIW treatment. The L-IGTGIPGIW-treated group significantly decreased glomerulus size by 1.61 ± 0.03-fold, and the H-IGTGIPGIW-treated group significantly reduced glomerulus size by 1.16 ± 0.04-fold, both compared to the SHR control. In addition, the SP-treated group also dramatically decreased the glomerulus size by 0.98 ± 0.05-fold compared to the SHR control.

### 3.5. Protective Effect of IGTGIPGIW on Hypertension-Induced Heart Damage in SHR

**Figure 4:**
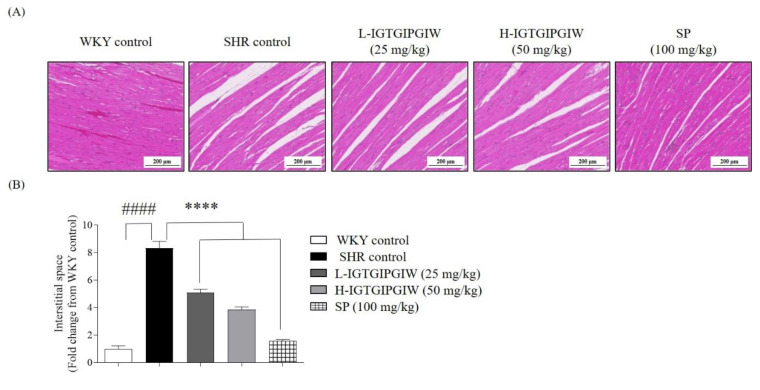
Effect of IGTGIPGIW on Pathomorphology of the myocardial tissues in spontaneously hypertensive rats (SHR). SHR at 8 weeks showed abnormalities in myocardial tissues, and increased interstitial spaces were observed in SHR. Histologic H&E staining of myocardial tissue (**A**) and interstitial spaces (**B**). Interstitial spaces were measured using ImageJ software (v.1.8.0). Data expressed as the mean ± SD. Significant differences were identified at **** *p* < 0.0001, as compared to the SHR control; #### *p* < 0.0001, as compared to the Wistar Kyoto rats (WKY) control.

Building on the vascular and renal findings, we next focused on the cardioprotective effects of IGTGIPGIW in SHR. To evaluate the potential cardioprotective properties of IGTGIPGIW, histological analysis was performed on heart tissues collected from SHR to assess morphological changes. As an indicator of structural myocardial damage associated with hypertension, special attention was given to evaluating the interstitial distance between cardiac muscle fibers. In Figure 4A, the heart tissue image revealed that the interstitial spaces were dramatically increased in SHR control compared with WKY control. The increased interstitial spaces of heart tissues were remarkably reduced in the IGTGIPGIW and SP-treated group. To assess the cardiac protective effects, we measured the interstitial spaces in heart tissues. In SHR control, the interstitial space increased by 8.33 ± 0.50-fold compared with WKY control, 1.00 ± 0.22-fold. However, IGTGIPGIW treatment notably diminished the interstitial space in heart tissues. The L-IGTGIPGIW group showed a significant 5.08 ± 0.27-fold reduction, while the H-IGTGIPGIW group exhibited a comparable decrease of 3.86 ± 0.19-fold. In line with these findings, SP treatment also led to a marked reduction of 1.58 ± 0.09-fold in interstitial spacing. These results indicated that IGTGIPGIW possessed cardioprotective effects in SHR.

## 4. Discussion

Seahorses are widely recognized as threatened worldwide due to intensive use in traditional medicine, the aquarium trade, and ecotourism [30]. However, aquaculture of the big-belly seahorse (*H. abdominalis*) has been successfully established in Australia and, more recently, on Jeju Island in Korea [31,32]. The successful establishment of big-belly seahorse aquaculture secures a stable and scalable biomass supply. The extensive research has been conducted to validate the functional ingredients and evaluate the potential biological activities of seahorses, to promote their application across diverse industries, particularly in pharmaceuticals and health functional food development. In our previous studies, extracts and bioactive peptides from *H. abdominalis* exhibited significant functional benefits, enhancing muscle strength [33], exerting antioxidant effects [34,35], regulating blood pressure [36], and conferring anti-fatigue activity [37], thereby substantiating their potential as candidates for therapeutic development and health functional food ingredients. Building on these findings, the present follow-up investigation further confirmed their antihypertensive efficacy in an in vivo study, thereby serving as preliminary research for the development of health functional foods with blood pressure regulatory efficacy. Long-term administration of IGTGIPGIW gradually reduced blood pressure, with no significant decrease observed at week 7; however, a significant SBP, DBP, and MAP blood pressure regulatory effect was evident at a dose of 100 mg/kg by week 8. Similarly, the positive control sardine peptide did not show a significant reduction in SBP, DBP, or MAP at week 7; however, a significant decrease was observed at week 8, and these values were comparable to those obtained with H-IGTGIPGIW. Treatment with H-IGTGIPGIW resulted in a marked reduction of blood pressure parameters, with SBP, DBP, and MAP decreased by approximately 40.90 mmHg, 52.22 mmHg, and 51.70 mmHg, compared with the SHR control group. In a previous study, the hydrolysates derived from sardine peptides also exhibited blood pressure regulatory efficacy, with a maximal reduction of SBP by approximately 13 mmHg after four weeks of administration [38]. In another study, purified fish proteins derived from salmon, tuna, cod, and a mixture of white fish demonstrated notable blood pressure regulatory efficacy, with a maximal reduction of SBP by approximately 30.9 mmHg (14% decrease compared to the casein-based diet) after eight weeks of administration [39]. These findings indicate that long-term administration of H-IGTGIPGIW exerted superior blood pressure-lowering efficacy, particularly in terms of SBP reduction, compared with previously reported fish-derived peptide or hydrolysate.

The ACE inhibitory activity of peptides is highly influenced by their amino acid composition and sequence characteristics, as specific residues play crucial roles in binding affinity and interaction with the active site of the enzyme. An earlier research study demonstrated that the hydrophobicity of peptides, as well as the presence of specific N-terminal and C-terminal residues, plays a crucial role in inhibiting ACE activity by enhancing the interaction between the peptide and the active site of ACE; in particular, a peptide containing an N-terminal leucine and a C-terminal proline effectively inhibits ACE activity by increasing hydrophobic interactions with the ACE receptor [40]. Therefore, we hypothesized that the peptide IGTGIPGIW, which possesses an N-terminal isoleucine also enhances binding affinity to the active site of ACE, thereby inhibiting its enzymatic activity and potentially contributing to the regulation of blood pressure.

Serum analysis revealed that treatment with H-IGTGIPGIW significantly decreased Ang Ⅱ and ACE levels while markedly increasing ACE2 levels in SHR. A similar trend was also observed in the positive control group, where sardine peptide treatment produced comparable effects on Ang Ⅱ and ACE levels [41]. In our previous study, hydrolysates derived from the red sea cucumber were also shown to regulate serum Ang Ⅱ, ACE, and ACE2 levels, thereby contributing to blood pressure regulation [42]. Therefore, IGTGIPGIW appears to bind strongly to the active site of ACE, thereby inhibiting its activity and consequently leading to a decrease in serum Ang Ⅱ and ACE levels. This inhibition suppresses the conversion of angiotensin I (Ang I) to angiotensin II (Ang Ⅱ), ultimately contributing to the reduction of blood pressure. Furthermore the activation of ACE2 in serum possesses blood pressure-lowering effects, thereby validating its potential as a therapeutic target for antihypertensive drug development [43]. Interestingly, H-IGTGIPGIW intake was found to activate ACE2 in serum, and previous research has suggested that elevated levels of soluble ACE2 may exert antiviral effects by intercepting viral particles such as SARS-CoV-2, thereby potentially reducing the risk of infection [44]. Taken together, these results indicate that the intake of IGTGIPGIW exerts blood pressure regulatory effects by significantly reducing Ang Ⅱ and ACE levels while markedly increasing ACE2 levels in SHR. Moreover, the elevation of soluble ACE2 in serum could potentially provide additional protective benefits by acting as a decoy for viral particles such as SARS-CoV-2, thereby possibly contributing to a reduced risk of infection.

To further elucidate the protective effects of H-IGTGIPGIW against hypertension, histological analyses were conducted to assess tissue damage in the aorta, kidney (renal tubules and glomeruli), and heart. Histological examination revealed that treatment with H-IGTGIPGIW significantly reduced aortic wall thickness compared with the SHR control group. Moreover, the aortic thickness observed in the H-IGTGIPGIW-treated group was comparable to that of the positive control group receiving sardine peptide. In our previous study, a blood pressure-regulatory peptide derived from olive flounder also significantly reduced aortic wall thickness compared with the SHR control group [45]. Je et al. (2024) [46] also demonstrated that a peptide derived from *Sebastes schlegelii* exhibited blood pressure-regulatory effects and significantly reduced aortic wall thickness in SHR. Our findings further demonstrated that in hypertensive SHR, sustained high blood pressure induces compensatory thickening of the aortic wall, which reduces its elasticity. However, treatment with the IGTGIPGIW peptide effectively prevented this pathological thickening, resulting in an aortic wall thickness comparable to that observed in the normotensive WKY group.

Subsequent histological analysis of the kidney revealed that treatment with IGTGIPGIW led to an expansion of the renal tubule area and an increased number of renal tubules per unit area, which was comparable to the results observed in the positive control group treated with sardine peptide, indicating its protective effects on hypertensive renal pathology. Similarly, peptides derived from pea clam were also shown to ameliorate renal tubular damage in SHR, supporting their protective role against hypertensive renal injury [47]. These results indicate that in SHR, renal fibrosis is accelerated, leading to irregular morphology of the renal tubules, enlargement due to tubular fusion caused by renal injury, and a reduction in the number of renal tubules. However, treatment with IGTGIPGIW effectively protected against these pathological changes, preserving renal tubular structure. In addition, histological analysis revealed that the glomerular size was significantly reduced in the IGTGIPGIW-treated group compared with the SHR control group, and the values were comparable to those observed in the positive control group treated with sardine peptide. Son et al. (2021) demonstrated that dieckol, isolated from *Ecklonia cava,* exerts protective effects against glomerular sclerosis, a pathological change commonly observed in hypertensive nephropathy of SHR [48]. In addition, Ware et al. (2021) confirmed that in hypertensive SHR, enlargement of the glomeruli is accompanied by an increased number of sclerosed glomeruli, ultimately leading to the progression of glomerulosclerosis [49]. Taken together, these findings suggest that IGTGIPGIW also protects renal glomeruli in SHR by suppressing glomerular enlargement, thereby demonstrating its protective efficacy against hypertensive renal injury.

Histological analysis of the heart further demonstrated that treatment with IGTGIPGIW reduced the interstitial distance between myocardial fibers, indicating its protective effects against hypertensive cardiac tissue damage. Similarly, Yang et al. (2019) [50] reported comparable findings in SHR, demonstrating that the cardiac myocyte area was increased under hypertensive conditions, whereas treatment with bioactive peptides significantly reduced the cardiac myocyte area and alleviated cardiac damage. Collectively, our findings demonstrate that IGTGIPGIW exerts protective effects against cardiac damage in SHR, further supporting its potential as a therapeutic peptide for hypertension-induced cardiac injury.

## 5. Conclusions

In conclusion, IGTGIPGIW, a bioactive peptide derived from *H. abdominalis*, exhibited potential blood pressure-regulatory effects by modulating Ang II and ACE levels while upregulating ACE2 expression in SHR. In addition, the peptide showed significant protective effects against hypertension-induced tissue damage, including aorta, kidney, and heart tissue injuries. Through the evaluation of the blood pressure-regulating efficacy of functional peptides present in *H. abdominalis* extract, this study provides fundamental research data. It highlights the potential for developing functional foods that regulate blood pressure and prevent vascular and cardiac damage associated with hypertension.

## Figures and Tables

**Table 1:** Effect of IGTGIPGIW on serum angiotensin II (Ang II), angiotensin-Ⅰ-converting enzyme (ACE), angiotensin converting enzyme 2 (ACE 2) in SHR.

Groups	Ang II (pg/mL)	ACE (ng/mL)	ACE 2 (ng/mL)
WKY control	1155.25 ± 48.04 ^#^	8.62 ± 0.02 ^####^	0.45 ± 0.07 ^##^
SHR control	1323.20 ± 15.76	9.46 ± 0.12	0.70 ± 0.03
L-IGTGIPGIW	1291.39 ± 5.85	8.88 ± 0.42 ****	0.69 ± 0.02
H-IGTGIPGIW	1209.75 ± 6.36 *	8.66 ± 0.32 ****	0.88 ± 0.02 ***
SP	1178.37 ± 9.93 **	8.63 ± 0.08 ****	0.96 ± 0.02 ***

Significant differences were identified at * *p* < 0.05, ** *p* < 0.01, *** *p* < 0.001, and **** *p* < 0.0001, as compared to the SHR control, and ^#^ *p* < 0.05, ^##^ *p* < 0.01 and ^####^ *p* < 0.0001, as compared to the WKY control. Spontaneously hypertensive rats (SHR); Wistar Kyoto rats (WKY); Sardine-derived peptide (SP).

## Data Availability

The original contributions presented in this study are included in the article. Further inquiries can be directed to the corresponding authors.

## References

[1] Carey R.M., Whelton P.K., 2017 ACC/AHA Hypertension Guideline Writing Committee (2025). Prevention, Detection, Evaluation, and Management of High Blood Pressure in Adults: Synopsis of the 2017 American College of Cardiology/American Heart Association Hypertension Guideline. Ann. Intern. Med..

[2] Da E., Pereira C., Ximenes L., Pires C.M. (2025). Analysis of Secondary Data Utilization for Hypertension Prevention in Maubara Community Health Centre, Liquiça Municipality. Int. J. Sci. Multidiscip. Res..

[3] Yusuf S., Joseph P., Rangarajan S., Islam S., Mente A., Hystad P., Brauer M., Kutty V.R., Rahman O., Zatonska K. (2020). Modifiable Risk Factors, Cardiovascular Disease, and Mortality and Low-Income Countries (PURE): A Prospective Cohort Study. Lancet.

[4] Sharp S.I., Aarsland D., Day S., Sønnesyn H. (2011). Hypertension Is a Potential Risk Factor for Vascular Dementia: Systematic Review. Int. J. Geriatr. Psychiatry.

[5] Debora C., Tolimba C., Palunggi S., Siregar D., Harefa L. (2023). Risk Factors for Hypertension Among Adults Living in A Rural Area, Minahasa. J. Keperawatan Indones..

[6] Chun Y., Ying S., Yin W., Wei C., Fei M. (2020). New Flavonoid-Based Compound Synthesis Strategy for Antihypertensive Drug Development. Life Sci..

[7] Kuang Z.M. (2020). Effect of Combined Antihypertensive and Lipid-Lowering Therapies on Cognitive Function: A New Treatment Strategy?. Cardiol. Res. Pract..

[8] Ellison D.H., Loffing J. (2009). Thiazide Effects and Adverse Effects Insights from Molecular Genetics. Hypertension.

[9] Pal D., Nayak A.K. (2020). Bioactive Natural Products for Pharmaceutical Applications.

[10] Kumar S., Andrade P.B., Nicosia A., Cuttitta A., Rotter A., Rotter A., Bacu A., Barbier M., Bertoni F., Bones A.M. (2020). A New Network for the Advancement of Marine Biotechnology in Europe and Beyond. Front. Mar. Sci..

[11] Kowalska H., Czajkowska K., Cichowska J., Lenart A. (2017). Trends in Food Science & Technology What’ s New in Biopotential of Fruit and Vegetable by-Products Applied in the Food Processing Industry. Trends Food Sci. Technol..

[12] Gajaria T.K., Suthar P., Baghel R.S., Balar N.B., Sharnagat P., Mantri V.A., Reddy C.R.K. (2017). Bioresource Technology Integration of Protein Extraction with a Stream of Byproducts from *Marine macroalgae*: A Model Forms the Basis for Marine Bioeconomy. Bioresour. Technol..

[13] Riquelme M.Z., Rodr D., Javier F., Rubilar O., Alvear M., Encina-montoya F., Vidal G. (2023). Composting as an Alternative for the Treatment of Solid Waste from the Kraft Pulp Industry. Agronomy.

[14] Ayilara M.S., Olanrewaju O.S., Babalola O.O. (2020). Waste Management through Composting: Challenges and Potentials. Sustainability.

[15] Schieber A., Stintzing F.C., Carle R. (2002). By-Products of Plant Food Processing as a Source of Functional Compounds—Recent Developments. Trends Food Sci. Technol..

[16] Kim S. (2014). Seafood Processing By-Products.

[17] Kim S., Wijesekara I. (2010). Development and Biological Activities of Marine-Derived Bioactive Peptides: A Review. J. Funct. Foods.

[18] Korczek K., Tkaczewska J., Migdał W. (2018). Antioxidant and Antihypertensive Protein Hydrolysates in Fish Products—A Review. Czech J. Food Sci..

[19] Neves A.C., Harnedy P.A., Kee M.B.O., Alashi M.A., Aluko R.E., Fitzgerald R.J. (2017). Peptide Identification in a Salmon Gelatin Hydrolysate with Antihypertensive, Dipeptidyl Peptidase IV Inhibitory and Antioxidant Activities. Food Res. Int..

[20] Yathisha U.G., Bhat I., Karunasagar I., Mamatha B.S. (2018). Antihypertensive Activity of Fish Protein Hydrolysates and Its Peptides. Food Sci. Nutr..

[21] Walquist M.J., Eilertsen K.-E., Elvevoll E.O., Jensen I.-J. (2024). Marine-Derived Peptides with Anti-Hypertensive Properties: Prospects for Pharmaceuticals, Supplements, and Functional Food. Mar. Drugs.

[22] Yi Y., Lv Y., Zhang L., Yang J., Shi Q. (2018). High Throughput Identification of Antihypertensive Peptides from Fish Proteome Datasets. Mar. Drugs.

[23] Zheng Y., Zhang Y., San S. (2020). Efficacy of a Novel ACE-Inhibitory Peptide from *Sargassum maclurei* in Hypertension and Reduction of Intracellular Endothelin-1. Nutrients.

[24] Udenigwe C.C., Mohan A. (2014). Mechanisms of Food Protein-Derived Antihypertensive Peptides Other than ACE Inhibition. J. Funct. Foods.

[25] Ra J.E., Woo S.Y., Jin H., Lee M.J., Kim H.Y., Ham H., Chung I.M. (2020). Evaluation of Antihypertensive Polyphenols of Barley (*Hordeum vulgare* L.) Seedlings via Their Effects on Angiotensin—Converting Enzyme (ACE) Inhibition. Appl. Biol. Chem..

[26] Long A.N., Dagogo-jack S. (2011). Comorbidities of Diabetes and Hypertension: Mechanisms and Approach to Target Organ Protection. J. Clin. Hypertens..

[27] Cipolla M.J., Liebeskind D.S., Chan S. (2018). The Importance of Comorbidities in Ischemic Stroke: Impact of Hypertension on the Cerebral Circulation. J. Cereb. Blood Flow. Metaboloism.

[28] Bozkurt B., Aguilar D., Deswal A., Dunbar S.B., Francis G.S., Horwich T., Jessup M., Kosiborod M., Pritchett A.M., Ramasubbu K. (2016). Contributory Risk and Management of Comorbidities of Hypertension, Obesity, Diabetes Mellitus, Hyperlipidemia, and Metabolic Syndrome in Chronic Heart Failure: A Scientific Statement from the American Heart Association. Circulation.

[29] Lee H.G., Kim H.S., An H., Baek K., Lee J.M., Yim M.J., Ko S.C., Kim J.Y., Oh G.W., Je J.G. (2022). Antihypertensive Effects of IGTGIPGIW Peptide Purified from *Hippocampus Abdominalis*: P-ENOS and p-AKT Stimulation in EA.Hy926 Cells and Lowering of Blood Pressure in SHR Model. Mar. Drugs.

[30] Leach H., Lin Q., Lu J., Gao Y., Shen L., Cai J., Luo J. (2019). Effects of Food, The Effect of Temperature on Gonad, Embryonic Development and Survival Rate of Juvenile Seahorses, Hippocampus Kuda Bleeker. Aquaculture.

[31] Koldewey H.J., Martin-smith K.M. (2010). Review Article A Global Review of Seahorse Aquaculture. Aquaculture.

[32] Muthuramalingam K., Ho J., You K., Jeon J., Rho S. (2017). Effects of Sea Horse (*Hippocampus abdominalis*)-Derived Protein Hydrolysate on Skeletal Muscle Development. Food Sci..

[33] Muthuramalingam K., Kim S., Kim Y., Kim H. (2019). Bigbelly Seahorse (*Hippocampus abdominalis*)-Derived Peptides Enhance Skeletal Muscle Differentiation and Endurance Performance via Activated P38MAPK/AKT Signalling Pathway: An In Vitro and In Vivo Analysis. J. Funct. Foods.

[34] Lee H., Nagahawatta D.P., Yang F., Jayawardhana H.H.A.C.K., Liyanage N.M., Lee D., Min J., Yim M., Ko S., Kim J. (2023). Antioxidant Potential of Hydrolysate-Derived Seahorse (*Hippocampus abdominalis*) Peptide: Protective Effects against AAPH-Induced Oxidative Damage In Vitro and In Vivo. Food Chem..

[35] Soo H., Jun K., Je G., Ryu B., Kang N., Fernando I.P.S., Jayawardena T.U., Sanjeewa K.K.A., Young J., Tae O. (2018). Antioxidant and Angiotensin-I Converting Enzyme Inhibitory Peptides from *Hippocampus Abdominalis*. Eur. Food Res. Technol..

[36] Je J., Kim H., Lee H., Oh J., An Y., Wang L., Rho S., Jeon Y. (2020). Low-Molecular Weight Peptides Isolated from Seahorse (*Hippocampus abdominalis*) Improve Vasodilation via Inhibition of Angiotensin-Converting Enzyme In Vivo and In Vitro. Process Biochem..

[37] Kang N., Kim S., Rho S., Ko J., Jeon Y. (2017). Anti-Fatigue Activity of a Mixture of Seahorse (*Hippocampus abdominalis*) Hydrolysate and Red Ginseng. Fish. Aquat. Sci..

[38] Huang J., Liu Q., Xue B., Chen L., Wang Y., Ou S., Peng X. (2016). Angiotensin-I-Converting Enzyme Inhibitory Activities and In Vivo Antihypertensive Effects of Sardine Protein Hydrolysate. J. Food Sci..

[39] Ait-yahia D., Madani S., Savelli J., Prost J., Bouchenak M., Belleville J. (2003). Dietary Fish Protein Lowers Blood Pressure and Alters Tissue Polyunsaturated Fatty Acid Composition in Spontaneously Hypertensive Rats. Nutrients.

[40] Ding Q., Sheikh A.R., Chen Q., Hu Y., Sun N., Su X. (2023). Understanding the Mechanism for the Structure–Activity Relationship of Food-Derived ACEI Peptides. Food Rev. Int..

[41] Luo J., Zhang C., Liu Q., Ou S., Zhang L., Peng X. (2017). Combinative Effect of Sardine Peptides and Quercetin Alleviates Hypertension Through Inhibition of Angiotensin I Converting Enzyme Activity and Inflammation. Food Res. Int..

[42] Lee H.-G., Nagahawatta D.P., Liyanage N.M., Choe Y.R., Oh J.-Y., Jung W.-K., Park S.-H., Jeon Y.-J., Kim H.-S. (2024). Potential Blood Pressure Regulatory Effect of Low Molecular Weight α-Chymotrypsin Extract and Its Peptides from *Stichopus japonicus*: Peptide–ACE Interaction Study via In Silico Molecular Docking. J. Funct. Foods.

[43] Hernández Prada J.A., Ferreira A.J., Katovich M.J., Shenoy V., Qi Y., Santos R.A.S., Castellano R.K., Lampkins A.J., Gubala V., Ostrov D.A. (2008). Structure-Based Identification of Small-Molecule Angiotensin-Converting Enzyme 2 Activators as Novel Antihypertensive Agents. Hypertension.

[44] Keeffe M.O., Oterhals Å., Anja L., Vikøren S., Drotningsvik A., Mellgren G., Halstensen A., Gudbrandsen O.A. (2023). Dietary Fish Intake Increased the Concentration of Soluble ACE2 in Rats: Can Fish Consumption Reduce the Risk of COVID-19 Infection through Interception of SARS-CoV-2 by Soluble ACE2?. Br. J. Nutr..

[45] Lee H., Oh J., Chung D., Seo M., Park S., Jeon Y., Ryu B. (2022). Utility of a Hydrolysate from Overproduced Paralichthys Olivaceus for Hypertension Treatment: Correlation Between Physical Properties and Potent Anti-Hypertensive Activities. Mar. Drugs.

[46] Je J.-G., Sim J., Lee H.-G., Kim C.-Y., Roh Y., Choe Y.R., Park S.-H., Heo S.-J., Jung W.-K., Jeon Y.-J. (2024). Investigation of the Regulatory Effect of α-Chymotrypsin-Assisted Hydrolysate from Sebastes schlegelii on Blood Pressure through In Vitro, In Silico ACE Inhibitory Activity, and In Vivo Spontaneously Hypertensive Rat Model. J. Funct. Foods.

[47] Sun X., Wang M., Xu C., Wang S., Li L., Zou S., Yu J., Wei Y. (2022). Positive Effect of a Pea—Clam Two-Peptide Composite on Hypertension and Organ Protection in Spontaneously Hypertensive Rats. Nutrients.

[48] Son M., Oh S., Choi J., Jang J.T., Son K.H., Byun K. (2021). Attenuating Effects of Dieckol on Hypertensive Nephropathy in Spontaneously Hypertensive Rats. Int. J. Mol. Sci..

[49] Ware K., Yildiz V., Xiao M., Medipally A., Hemminger J., Scarl R., Satoskar A.A., Hebert L., Ivanov I., Biederman L. (2021). Hypertension and the Kidney: Reduced Kidney Mass Is Bad for Both Normotensive and Hypertensive Rats. Am. J. Hypertens..

[50] Yang C., Nithiyanantham S., Ying J. (2019). Bioactive Peptides Attenuate Cardiac Hypertrophy and Fibrosis in Spontaneously Hypertensive Rat Hearts. J. Food Drug Anal..

